# Driver mutations of cancer epigenomes

**DOI:** 10.1007/s13238-014-0031-6

**Published:** 2014-03-14

**Authors:** David M. Roy, Logan A. Walsh, Timothy A. Chan

**Affiliations:** 1 Human Oncology and Pathogenesis Program, Memorial Sloan-Kettering Cancer Center, New York, NY 10065 USA; 2 Weill Cornell/Rockefeller/Sloan-Kettering Tri-Institutional MD-PhD Program, New York, NY 10065 USA; 3 Department of Radiation Oncology, Memorial Sloan-Kettering Cancer Center, New York, NY 10065 USA

**Keywords:** chromatin, cancer, epigenetics, mutations, methylation

## Abstract

Epigenetic alterations are associated with all aspects of cancer, from tumor initiation to cancer progression and metastasis. It is now well understood that both losses and gains of DNA methylation as well as altered chromatin organization contribute significantly to cancer-associated phenotypes. More recently, new sequencing technologies have allowed the identification of driver mutations in epigenetic regulators, providing a mechanistic link between the cancer epigenome and genetic alterations. Oncogenic activating mutations are now known to occur in a number of epigenetic modifiers (i.e. *IDH1*/*2*, *EZH2*, *DNMT3A*), pinpointing epigenetic pathways that are involved in tumorigenesis. Similarly, investigations into the role of inactivating mutations in chromatin modifiers (i.e. *KDM6A*, *CREBBP*/*EP300*, *SMARCB1*) implicate many of these genes as tumor suppressors. Intriguingly, a number of neoplasms are defined by a plethora of mutations in epigenetic regulators, including renal, bladder, and adenoid cystic carcinomas. Particularly striking is the discovery of frequent histone H3.3 mutations in pediatric glioma, a particularly aggressive neoplasm that has long remained poorly understood. Cancer epigenetics is a relatively new, promising frontier with much potential for improving cancer outcomes. Already, therapies such as 5-azacytidine and decitabine have proven that targeting epigenetic alterations in cancer can lead to tangible benefits. Understanding how genetic alterations give rise to the cancer epigenome will offer new possibilities for developing better prognostic and therapeutic strategies.

## INTRODUCTION

Cancer is an evolutionary process through which dysregulation in select cellular mechanisms confers a clonal advantage, leading to tumor growth and eventually, metastasis. In fact, nearly all cancers are defined by several “hallmark” capabilities, including resisting cell death, evading growth suppressors, uncontrolled proliferation, neoangiogenesis, invasion/metastasis, and replicative immortality (Hanahan and Weinberg, 2011). Aberrant control of other processes, such as defective differentiation and DNA damage repair, is also linked to tumor formation. Research efforts have traditionally focused on genetic abnormalities underlying malignant transformation, due to initial technological constraints and our limited understanding of other heritable patterns of gene regulation. Early studies on these genetic alterations—copy number variations, mutations, gene rearrangements—have defined mechanisms of oncogenesis, led to the creation of targeted therapies, and improved patient outcomes for certain cancers. Only recently has it become evident that many genetic alterations in cancer target epigenetic regulators, causing cancer-associated phenotypes via epigenetic dysfunction.

**Figure 1:**
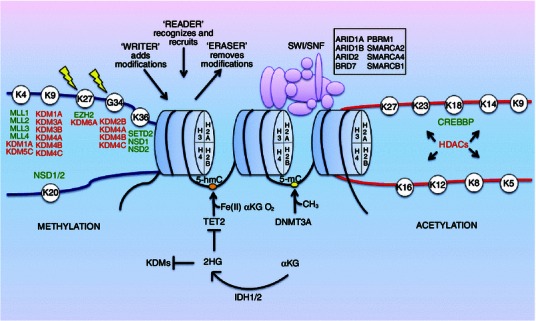
**Epigenetic regulation of gene expression**. Epigenetic processes include DNA methylation, covalent histone modifications (e.g. methylation, acetylation), and chromatin remodeling (SWI/SNF complex). Modifier proteins with frequent driver mutations in cancer are shown by specific function and target site. Green and red font colors represent histone “writers” and “erasers,” respectively. Lightning bolts represent cancer-associated mutations in histones H3.1 (K27) and H3.3 (K27, G34)

Since the first discovery of cancer-associated loss of DNA methylation, the field of cancer epigenetics has grown remarkably and helped elucidate aspects of cancer biology where genetic explanations alone are insufficient (Feinberg and Vogelstein, 1983). Epigenetics is the process by which cells encode non-genetic, heritable information through alterations that do not change the DNA sequence. Generally, chromatin exists in two main forms—condensed, transcriptional silent heterochromatin and euchromatin, which is transcriptionally active. The functional unit of chromatin is the nucleosome, which is an octameric structure composed of two histones each of H2A, H2B, H3, and H4 encircled by 147 bp of DNA (Margueron and Reinberg, 2010). Regulation of the chromatin state is achieved through DNA methylation, chromatin remodeling, and/or covalent histone modifications, such as methylation, acetylation, phosphorylation, ubiquitination, and sumoylation. The major effectors of these modifications are the chromatin modifier enzymes, highly specific proteins that catalyze the addition or removal of functional groups to DNA or histone tails. These modifications alter chromatin structure through noncovalent interactions within and between nucleosomes, leading to changes in macromolecular organization and promoter accessibility. In addition, these chromatin “marks” serve as signals to other specialized proteins involved in chromatin organization, gene transcription, genome maintenance, and replication (Kouzarides, 2007; Sharma et al., 2010). As a result, aberrations in one or more of these modifiers can have profound effects on normal cell physiology and are now well-documented in many diseases, including cancer (Fig. 1).

It has been known for decades that epigenetic dysregulation occurs in cancer. Aberrant DNA hyper- and hypo-methylation was one of the first molecular features noted to be present in cancer cells (Feinberg and Vogelstein, 1983; Gama-Sosa et al., 1983; Rubery and Newton, 1973; Timp and Feinberg, 2013). However, the nature of epigenetic alterations and the molecular underpinnings of these changes are just beginning to be appreciated. The recent accelerated search for mutations in cancers, facilitated through next generation sequencing technologies, has greatly accelerated our understanding of the mechanisms underlying epigenetic dysfunction in cancers.

Genome-wide and exomic sequencing data from recent years have demonstrated that mutations in epigenetic modifiers comprise a large portion of all genetic events in many cancers, including tumors such as renal carcinoma, adenoid cystic carcinoma (ACC), and transitional cell carcinoma (TCC) of the bladder (Dalgliesh et al., 2010; Gui et al., 2011; Ho et al., 2013). In fact, the main objective of these recent genomic analyses is the identification of *bona fide* “driver” mutations in cancer genes. These mutations are defined by their ability to promote or “drive” tumorigenesis and are therefore positively selected for in the development of cancer. In contrast, “passenger” mutations, which comprise the majority of mutations identified, represent genetic events that have no direct or indirect effect on the selective growth advantage of the cell in which it occurred (Stratton et al., 2009; Vogelstein et al., 2013). Candidate epigenetic driver genes have been identified as either mutated across many cancers (e.g. *KDM6A* in over 12 cancers), having highly recurrent mutations (e.g. *IDH1*R132), or having highly prevalent mutations in select tumor histologies (e.g. *MLL2* in follicular lymphoma) (You and Jones, 2012). The characterization of these driver gene mutations has enhanced our understanding of the mechanisms contributing to oncogenesis, allowed for improved prognostic assessment, and opened the door for the development of exciting new therapies. In this review, we highlight recent developments, new discoveries, and therapeutic advances involving cancer-associated mutations in epigenetic regulators.

## DNA METHYLATION AND HYDROXYMETHYLATION

DNA methylation plays a well-defined role in both development and disease, including cancer. First identified in 1975, CpG island (CGI) methylation was shown to function as a relatively stable alteration on DNA that can serve to silence gene transcription (Holliday and Pugh, 1975; Riggs, 1975). We now understand that DNA methylation is much more dynamic and complex, with diverse epigenetic consequences linked to varied genomic locations of where this mark occurs. For example, DNA methylation at gene promoter CGIs potently blocks the initiation of transcription, whereas methylation within CpG-poor gene bodies may actually facilitate elongation and influence patterns of alternate splicing. In addition, DNA methylation is frequently found in repeat-rich areas of the genome and is vital for both chromosomal and genomic stability, possibly through the repression of retroviral transposons (Jones, 2012). Still, the role for this epigenetic mark at other regulatory regions, such as enhancers and insulators, has yet to be determined. Regardless, aberrant methylation in human cancer is a defining feature, with global promoter CGI hypermethylation and non-CGI hypomethylation widely reported (Ehrlich, 2002; Sharma et al., 2010). Furthermore, local variations in methylation at only several key loci have been shown to be sufficient for tumorigenesis (Lee et al., 2008; Poage et al., 2011). Importantly, these altered patterns of DNA epigenetic marks (e.g. 5-mC, 5-hmC) are frequently accompanied by a critical imbalance in transcriptional programs involving differentiation and stem cell maintenance, thereby initiating tumorigenesis and sustaining growth (Jones and Baylin, 2007).

DNA methylation can function to silence tumor suppressor genes along with genetic mutations (Herman and Baylin, 2003). For example, in the case of hereditary gastric cancer, methylation of *CDH1* (which encodes the E-cadherin tumor suppressor) can function as a “second hit” and cause gastric cancer when the first allele is mutated (Grady et al., 2000). In sporadic cancers, tumor suppressor genes that are mutated in hereditary versions of the disease are frequently silenced by DNA methylation instead (Esteller et al., 2001). For example, in hereditary nonpolyposis colon cancer (HNPCC), *MLH1* inactivation via mutation can lead to microsatellite instability (MSI) and tumorigenesis, whereas in sporadic colon cancers, *MLH1* is frequently silenced by methylation (Kane et al., 1997; Veigl et al., 1998). These data and others indicate that aberrant DNA methylation can work along with genetic alterations to promote tumorigenesis.

### DNMT3A

DNA methylation is carried out by the mammalian DNA methyltransferases (DNMTs), essential enzymes that catalyze the addition of a methyl group to cytosine in CpG dinucleotides in DNA (Jones, 2012). The conversion of 5-cytosine (5-C) to methyl-cytosine (5-mC) requires the presence of a methyl donor, S-adenosylmethionine (SAM), and one of the following catalytically active DNMTs: DNMT1, DNMT3A, or DNMT3B (Shen et al., 1992). Although there is some evidence for overlapping roles, DNMT3A and DNMT3B are essential for *de novo* DNA methylation, whereas DNMT1 “maintains” heritable methylation patterns across the genome during cell replication (Hsieh, 1999). In fact, the role of DNMT3A in *de novo* methylation was initially characterized in the context of epigenetic silencing during development, including at imprinted loci in germ cells (Kaneda et al., 2004; Okano et al., 1999). The DNMTs have long been suspected to play a role in oncogenesis, as well. Aberrations in DNA methylation—both hyper- and hypo-methylation—have been well-documented in patient tumors and cell lines for decades (El-Osta, 2004). Additionally, Oka and colleagues first showed that in some cases, DNMT3A and DNMT3B, not DNMT1, mediate the cytotoxic effects of 5-aza-dC, a therapeutic mainstay in the treatment of several hematopoietic malignancies (Oka et al., 2005; Plimack et al., 2007). Not long after, DNMT3A and DNMT3B were further implicated in both hematopoietic stem cell (HSC) renewal and differentiation, two tightly regulated processes whose perturbation can lead to carcinogenesis (Challen et al., 2012; Tadokoro et al., 2007).

*DNMT3A* somatic mutations were first discovered by Yamashita and colleagues following sequencing of tissue from adult patients with *de novo* acute myeloid leukemia (AML) (Yamashita et al., 2010). Soon after, *DNMT3A* mutations were reported in AML cohorts from others, with frequencies as high as 22.1% (Ley et al., 2010). The majority of mutations occur at R882 (60%–64%), and almost all are heterozygous (Ley et al., 2010; Thol et al., 2011a). *DNMT3A* mutations are enriched in AML patients with intermediate-risk cytogenetics and normal karyotype (Lin et al., 2011; Patel et al., 2012). They are also associated with increased age, M4 and M5 AML subtypes, worse overall survival (OS) and relapse-free survival (RFS), and increased blasts at diagnosis (Hou et al., 2012; Ley et al., 2010; Lin et al., 2011; Marcucci et al., 2012; Thol et al., 2011a; Yan et al., 2011). There is also evidence that *DNTM3A* mutations may be a prognostic marker for decreased time to treatment failure (TTF), duration of complete remission (CR), and disease-free survival (DFS), at least in cytogenetically normal AML (Marcucci et al., 2012; Thol et al., 2011a; Yan et al., 2011). These studies have also identified several frequently co-occurring mutations, which include *NPM1*, *FLT3*, *IDH1*/*2*, and less commonly, *TET2* (Hou et al., 2012; Ley et al., 2010; Lin et al., 2011; Marcucci et al., 2012; Renneville et al., 2012; Thol et al., 2011a; Yan et al., 2011). As alterations in epigenetic regulators frequently lead to genomic instability, *DNMT3A* mutation may further drive progression of the disease itself (Wakita et al., 2013). Further, recent experimental work has demonstrated that DNMT3A mutations at R882 are only found in major clones, suggesting this genetic alteration may be an initiating lesion in AML (Bisling et al., 2013).

*DNMT3A* mutations have also been described, albeit at lower frequency, in other myeloid malignancies, such as myelodysplastic syndromes (MDS; 3%–8%) and myeloproliferative neoplasms (MPNs; 2%–10%), as well as early T-cell precursor acute lymphoblastic leukemia (ETP-ALL; 16%–18%) (Brecqueville et al., 2011; Ewalt et al., 2011; Grossmann et al., 2013; Neumann et al., 2013; Stegelmann et al., 2011; Thol et al., 2011b; Traina et al., 2013; Walter et al., 2011). Similar to what is observed in *de novo* AML, R882 is most frequently targeted for mutation (60%) in these neoplasms (Thol et al., 2011b). Clinically, *DNMT3A* mutations also correlated with increased age and predicted prognosis in all types, including worse OS, event-free survival (EFS), and AML-free survival (Lin et al., 2011; Neumann et al., 2013; Renneville et al., 2012; Thol et al., 2011b; Walter et al., 2011). Paradoxically, univariate and multivariate analysis of 92 patients with MDS revealed *DNMT3A* mutations were correlated with better overall response rate (ORR) and progression-free survival (PFS) (Traina et al., 2013). Interestingly, similar to AML, MPNs showed an association between *DNMT3A* alterations and mutation in *JAK2*, *IDH1*/*2*, and *ASXL1* but not *TET2* (Stegelmann et al., 2011). However, studies in MDS and ETP-ALL have found no association between *DNMT3A* mutations and other known leukemogenic drivers, including *FLT3* (Neumann et al., 2013; Thol et al., 2011b).

DNMT3A is a 102 kDa protein with three highly conserved functional domains: An N-terminal PWWP domain, a cysteine-rich PHD zinc finger domain, and a C-terminal catalytic domain (Hermann et al., 2004). *DNMT3A* mutations in cancer have been reported in all three domains, with most occurring in the catalytic domain, including the R882 mutation (60%) (Ley et al., 2010). Still, many cancer-specific mutations occur in non-catalytic domains. The PWWP domain is essential in localizing DNMT3A to heterochromatic regions of DNA during interphase, though it is unclear if this is related to its reported ability to bind DNA directly (Bachman et al., 2001; Ge et al., 2004; Purdy et al., 2010; Suetake et al., 2011). Alternatively, the PHD domain has also been shown to mediate regional specificity and repression through its interactions with transcriptional repressor RP58, HP1beta, histone deacetylatases (HDACs), and SUV39H1 (Datta et al., 2003; Fuks et al., 2001; Fuks et al., 2003). Exactly how these mutations disrupt protein function is of great interest, although a single unifying mechanism is unlikely to exist. Likewise, a recent clinical study found that R882 mutations confer poor prognosis in older populations, whereas non-R882 mutations confer poor prognosis in younger patients (Marcucci et al., 2012).

Whether these reported mutations are loss-of-function, gain-of-function, or act via a dominant-negative mechanism has also been debated. A strong case can be made that *DNMT3A* is an oncogene, as it is overexpressed in several cancers, depletion results in decreased proliferation and metastasis, and 5-aza-dC causes apoptosis through direct inhibition of DNMT3A (Oka et al., 2005). In regards to mutations themselves, the R882H DNMT3A mutant was sufficient to promote tumorigenesis in an IL-3 dependent transformation assay in leukemic 32D cells (Yan et al., 2011). Alternatively, some studies have demonstrated that mutant DNMT3A (R882H) has a markedly reduced catalytic ability (~50%) in methyltransferase assays and decreased DNA-binding capacity *in vitro*, implying a possible loss-of-function phenotype via a dominant-negative mechanism (Jia et al., 2007; Ley et al., 2010; Yamashita et al., 2010). Clearly, additional studies are necessary to understand the exact nature by which these cancer-associated mutations are transforming.

Characterizing larger DNMT3A-induced changes in the cancer methylome has proven quite challenging. Ley and colleagues showed no changes in genome-wide methylation according to *DNMT3A* status in AML, and although 182 specific regions showed increased hypomethylation in mutant samples, this did not correlate with gene expression (Ley et al., 2010). However, in a more recent cohort, a total of 3878 genomic regions were found to have significantly different methylation patterns using MeDIP-chip and differences of expression levels in 889 of 20,723 annotated genes was observed via an Affymetrix microarray (Yan et al., 2011). Further, this group found during RT-PCR validation that the expression of several HOX family genes significantly increased in DNMT3A mutant samples compared to wild-type.

Recent experiments may offer some insight behind the conflicting methylation and functional data. Protein binding at the DNMT3A tetramerization interface is important for methylation patterning, inducing processive methylation of clustered sites (Jia et al., 2007). Most mutations, including R882, occur within this tetramer interface. Therefore, differences between oligomerization states can explain how *DNMT3A* mutations alter epigenetic silencing and lead to transformation, without global changes in DNA methylation (Holz-Schietinger et al., 2011; Holz-Schietinger et al., 2012). Although the commonly occurring R882H mutation does not disrupt DNMT3A association with required cofactor DNMT3L *in vitro*, the latter is only expressed in early development (Chedin et al., 2002; Jia et al., 2007; Webster et al., 2005; Yamashita et al., 2010). However, other binding proteins, such as thymine-DNA glycosylase and ecotropic viral intergration site 1, can adhere to sites within the DNMT3A catalytic domain and may explain altered mutant DNMT3A activity (Li et al., 2007; Senyuk et al., 2011). In addition, non-catalytic mutations may disrupt protein binding to other domains, as described above. Although the exact mechanism remains elusive, these mutations result in decreased methylation processivity and altered localization, possibly to euchromatic regions of DNA (Jurkowska et al., 2011). This may help explain how *DNMT3A* mutations drive tumor formation in hematopoietic malignancies, even in the absence of larger global methylation changes.

### TET2

The ten-eleven translocation (TET) family proteins were first discovered in cancer with the fusion of TET1 to MLL in select AML patients with t(10;11)(q22;q23) (Lorsbach et al., 2003). Mechanistic studies then showed the TET proteins are dioxygenases that depend on 2-oxoglutarate, oxygen, Fe(II), and ascorbic acid to catalyze the conversion of 5-mC to 5-hydroxymethylcytosine (5-hmC) at CpG regions in DNA (Blaschke et al., 2013; Ito et al., 2010; Minor et al., 2013; Tahiliani et al., 2009; Yin et al., 2013). TET enzymes may be responsible for DNA demethylation through both passive and active means. For example, CpG dinucleotides that are “marked” with 5-hmC are not recognized by DNMT1 and therefore, methylation is passively lost at these sites through repeated cycles of cell division (Valinluck and Sowers, 2007). Alternatively, active demethylation can proceed following placement of 5-hmC via the activation-induced cytidine deaminase (AID)-APOBEC DNA repair pathway (Guo et al., 2011). More recently, an even greater role for TET enzymes in active demethylation was shown *in vitro*, with TET enzymes proving sufficient for converting 5-mC to 5-hmC, 5-hmC to 5-formylcytosine (5-fC), and finally 5-fC to 5-carboxylcytosine (5-caC). 5-caC is then targeted by base excision repair enzymes to complete the demethylation process (He et al., 2011; Ito et al., 2011).

*TET2* was first suspected to have a role in cancer when six patients with either secondary AML (sAML) or MDS were noted to have minimal deletions via FISH on chromosome 4q24 (Viguie et al., 2005). Soon after, the first *TET2* somatic mutations were identified in 25 patients (14%) with MPNs (Delhommeau et al., 2008). Delhommeau and colleages then sequenced *TET2* in patient tumor samples, becoming the first group to identify *TET2* mutations in multiple myeloid neoplasms, including MDS (19%), MPNs (12%–14%), and sAML (24%). They concluded that *TET2* was a novel *bona fide* tumor suppressor, noting that the majority of mutations are heterozygous (55%) and that *TET2* defects precede the well-known JAK2 V617F driver mutation in MPN HSCs (Delhommeau et al., 2009). More recently, Schaub and colleagues disputed this result using colony formation assays to show that *TET2* mutations can either precede (4 of 8 patients), follow (2 of 8), or occur independently (2 of 8) of JAK2 V617F mutations in MPN patient samples (Schaub et al., 2010). Although the temporal relationship between *TET2* mutations and other leukemogenic drivers is still unclear, the frequency and ubiquitous nature of these mutations in cancer is quite revealing. In addition to MDS, sAML, and MPNs, *TET2* mutations have now been described in other myeloid neoplasms, such as *de novo* AML (12%) and chronic myelomonocytic leukemia (CMML; 42%–46%) (Abdel-Wahab et al., 2009; Smith et al., 2010).

Unlike *DNMT3A* mutations, *TET2* alterations seem to hold limited prognostic utility in leukemia. The overwhelming majority of studies published to date have found no change in OS or any other prognostic tools between patients harboring *TET2* mutations and those who are not. However, one study in a cohort of 96 MDS patients reported that *TET2* mutations conferred an OS, EFS, and AML-free survival advantage (Kosmider et al., 2009). Paradoxically, Abdel-Wahab and colleagues found that *TET2* mutations were linked to worse OS in 93 patients with *de novo* AML, and a recent whole-exome sequencing study reported worse EFS in AML patients with *TET2* mutation (Abdel-Wahab et al., 2009; Weissmann et al., 2012). Further, Nibourel and colleagues reported no association with OS or DFS in their cohort of *de novo* AML patients, though the prevalence of *TET2* mutations in patients who failed to achieve complete remission (CR) trended higher (27% vs. 17%) (Nibourel et al., 2010). Despite questionable association to patient outcomes, *TET2* mutations are linked with other clinical features, including monocytosis, leukocytosis, and advanced age at diagnosis (Jankowska et al., 2009; Smith et al., 2010; Tefferi et al., 2009a; Tefferi et al., 2009c). Although *TET2* mutations show little association to known myeloid leukemogenic drivers*—FLT3-ITD, RUNX1, CEBPA—*they do associate with *NPM1* and *ASXL1* mutations and infrequently co-occur with *IDH1* or *IDH2* mutations (Chou et al., 2011a; Weissmann et al., 2012). Lastly, despite some data indicating no association between cytogenetics and *TET2* status in MPN, *TET2* mutations occur more often in the presence of normal karyotype and intermediate-risk AML (Hussein et al., 2010; Weissmann et al., 2012). In this cytogenetic setting, *TET2* mutations do predict significantly worse OS in AML (Chou et al., 2011a).

Although few *TET2* recurrent mutations have been reported, many mutations result in a frameshift or early stop codon and are therefore inactivating (Tefferi et al., 2009b). In fact, the largest proportion of nonsense mutations occur in exon 3, resulting in a truncated protein lacking the C-terminal catalytic domain (Moran-Crusio et al., 2011). Additionally, several missense mutations have been characterized as loss-of-function, with Ko and colleagues reporting impaired hydroxylation of 5-mC when mutant TET2 was overexpressed in HEK-293T cells. Furthermore, *TET2* mutation status is significantly correlated with decreased global 5-hmC in myeloid tumors (Ko et al., 2010; Konstandin et al., 2011). Functional studies manipulating *TET2* have also been able to recapitulate phenotypes that are characteristic of myeloid neoplasms, suggesting that *TET2* loss may be a key event in leukemic transformation. For example, a conditional mouse model for *TET2* loss in the hematopoietic compartment resulted in increased HSC self-renewal and myeloproliferation including splenomegaly, monocytosis, and extramedullary hematopoiesis (Moran-Crusio et al., 2011). This is consistent with other studies showing that *TET2* inactivation leads to decreased 5-hmC in HSCs, amplification of the stem cell population, and may skew HSCs toward a myeloid lineage (Pronier et al., 2011; Quivoron et al., 2011).

The true effect of *TET2* mutations on DNA methylation status has been difficult to ascertain. Despite an expected increase in 5-mC following *TET2* inactivation, several studies have reported a global decrease in methylation (Ko et al., 2010; Yamazaki et al., 2012). However, analysis of specific gene promoters shows mixed results in TET2 mutant samples, frequently exhibiting promoter-specific hypermethylation in spite of global hypomethylation (Perez et al., 2012; Wu et al., 2011; Yamazaki et al., 2012). Still, Ko and colleagues noted that several AML patients with wild-type TET2 had 5-hmC levels very similar to those patients with mutant TET2 (Ko et al., 2010). This suggests a more complex relationship between *TET2*, DNA methylation status, and malignant transformation.

In the past year, exciting new evidence has emerged to suggest a more diverse role for *TET2* in epigenetic regulation. In addition to known associations with polycomb repressive complex (PRC) regulator SIN3A and NURD complex member MBD3 (Wu et al., 2011; Yildirim et al., 2011), TET2 was recently identified as a direct binding partner with O-linked beta-N-acetylglucosamine transferase (OGT), an enzyme that marks histone H2B S112 at active transcription start sites (TSS) (Chen et al., 2013b). Although OGT doesn’t influence TET2 activity in functional assays, TET2 seems to actively target OGT to unmethylated promoters and activate transcription via other means (Vella et al., 2013). Furthermore, Deplus and colleagues showed that TET2 and OGT co-localize at active promoters marked by H3K4me3 through a direct interaction with host-cell factor 1 (HCF1) and that knockdown of *TET2* leads to global decreases of H3K4me3 and GlcNAcylation (Deplus et al., 2013). Another direct interaction has been described between TET2 and EBF1, a transcription factor that is associated with transcriptional activation and “poised” chromatin (Guilhamon et al., 2013; Treiber et al., 2010). Additional binding partners have also been reported within the past year, including NANOG and IDAX (Costa et al., 2013; Ko et al., 2013). Lastly, several novel miRNAs were discovered to negatively regulate *TET2* expression, offering a possible explanation for *TET*-associated transformation in the absence of any genomic alterations (Cheng et al., 2013; Fu et al., 2013; Song et al., 2013). Collectively, evidence is mounting that *TET2* inactivation in cancer may alter more than just DNA methylation; in fact, transformation may result considerably from disrupted interactions with other epigenetic regulators and development-associated transcription factors.

### IDH1/2

Isocitrate dehydrogenase 1 (IDH1) and isocitrate dehydrogenase 2 (IDH2) are two homodimeric metabolic enzymes that convert isocitrate to α-ketoglutarate (α-KG) while reducing NADP^+^ to NADPH. IDH1 is present in the cytosol and peroxisomes whereas IDH2 is located exclusively in mitochondria (Geisbrecht and Gould, 1999; Winkler et al., 1986; Xu et al., 2004). Frequent recurrent mutations in *IDH1* were initially discovered in GBM (12%) following whole-exome sequencing of 22 patient tumor samples (Parsons et al., 2008). Further sequencing efforts revealed that mutations are most prevalent in WHO grade II/III gliomas (71%) and secondary GBMs (88%) but less common in primary GBMs (7%) (Balss et al., 2008). Subsequent studies have shown that *IDH2* mutations are also enriched in WHO grade II/III gliomas, albeit less frequently, and that *IDH1*/*2* mutations occur in a mutually exclusive manner (Hartmann et al., 2009). These data indicate *IDH* mutation is an early event in glioma oncogenesis, frequently preceding known alterations like *TP53* mutation and 1p/19q loss (Watanabe et al., 2009). Interestingly, recent data suggest *IDH1* and *IDH2* mutations may actually differentially associate with astrocytoma and oligodendrogliomas, respectively (Hartmann et al., 2009). *IDH* mutations are associated with *MGMT* promoter hypermethylation, *TP53* mutation, 1p/19q codeletion, *ATRX* inactivation, younger age, and improved prognosis while being inversely correlated with *EGFR* amplification in glioma (Chou et al., 2010; Wiestler et al., 2013; Yan et al., 2009; Zou et al., 2013). Further, although early studies could not find any *IDH* mutations in other types of solid tumors (Bleeker et al., 2009; Kang et al., 2009), recurrent mutations have since been identified in chondrosarcoma (56%), cholangiocarcinoma (23%), melanoma (10%), and prostate cancer (2%) (Amary et al., 2011; Borger et al., 2012; Ghiam et al., 2012; Shibata et al., 2011).

Soon after the discovery of *IDH* mutations in glioma, recurrent mutations of *IDH* were also identified in AML (Green and Beer, 2010; Mardis et al., 2009). Similar to glioma, *IDH1* and *IDH2* mutations are mutually exclusive, though the mutational frequency of *IDH* in AML is much lower (23%) (Chou et al., 2011b; Ward et al., 2010). In contrast, the utility of *IDH* mutation status as an independent prognostic marker in AML is less clear. In a convincing cohort of 493 adult patients with AML, Chou and colleagues found that *IDH1* mutation had no impact on OS (Chou et al., 2010). Still, other studies have suggested a more disparate role, with mutation in *IDH1* and *IDH2* conferring poor and improved prognosis, respectively (Chou et al., 2011b; Schnittger et al., 2010). Notably, although another large cohort of 805 patients found that *IDH* mutation did not correlate with prognosis, a subset of patients with *IDH* mutation and CN-AML, *NPM1* mutation, and no *FLT3-ITD* did show significantly reduced OS and RFS (Paschka et al., 2010). Therefore, it is likely that the use of prognostic subsets—*IDH* status along with other genetic markers—may improve the utility of *IDH* status as a biomarker in AML. Other features that correlate with *IDH* mutation status include normal karyotype, intermediate-risk cytogenetics, *NPM1* mutation, and M1 AML subtype (Chou et al., 2010; Schnittger et al., 2010).

To date, practically all *IDH* mutations found in cancer are heterozygous and highly recurrent. Amino acid substitutions at residues IDH1-R132, IDH2-R172 and IDH2-R140Q are the most common, with considerable variability at R132 (R/H, R/C, R/S, R/G, R/L, R/V) and R172 (R/G, R/M, R/K) (Balss et al., 2008; Yan et al., 2009). The remarkable absence of any frameshift or nonsense mutations, deletions, or epigenetic silencing provided early evidence that *IDH* mutations were activating (Flanagan et al., 2012; Zhao et al., 2009). The R132 residue is located in the active site of IDH1 where it forms 2 hydrogen bonds with α- and β- carboxylate of isocitrate, its substrate (Nekrutenko et al., 1998; Xu et al., 2004). Initially, it was believed that these mutations may be loss-of-function or dominant-negative, as mutant IDH showed a reduced affinity for isocitrate and produced markedly less α-KG and NADPH *in vitro* (Yan et al., 2009; Zhao et al., 2009). However, an *in vitro* metabolite screen revealed that *IDH* mutations are neomorphic, producing the novel oncometabolite 2-hydroxyglutarate (2-HG) through heterodimer formation with the remaining wild-type IDH1. This was also verified in patient samples, with a strong correlation between the amount of 2-HG in tumor tissue and *IDH1*/*2* mutation status (Dang et al., 2009; Ward et al., 2010). In fact, 2-HG levels are increased 10–100 fold in patient sera and can be used to reliably diagnose *IDH* status and monitor response to therapy, though this application may be restricted to myeloid neoplasms (Capper et al., 2012; DiNardo et al., 2013; Ward et al., 2010).

The effects of mutant IDH are pleiotropic and affect numerous cell processes including DNA methylation, histone methylation, HIF1a signaling, collagen synthesis, and metabolism (Cairns and Mak, 2013). Remarkably, α-KG levels are unchanged in mutant *IDH* AML and glioma (Dang et al., 2009; Gross et al., 2010), and it is now clear that 2-HG-mediated inhibition of 2-OG-dependent dioxygenases is the dominant mechanism by which *IDH* mutations are oncogenic (Xu et al., 2011). Early data from the Cancer Genome Atlas project first identified the glioma hypermethylator phenotype (G-CIMP) in GBM and its association with *IDH* mutations (Laffaire et al., 2011; Noushmehr et al., 2010). Following this, Figueroa and colleagues demonstrated that 2-HG inhibition of the α-KG-dependent enzyme TET2 actively generates the hypermethylator phenotype in AML. Further, they showed that *TET2* and *IDH* mutations are mutually exclusive in AML, result in overlapping methylation signatures, and impair HSC differentiation in 32D myeloid cells (Figueroa et al., 2010). Similarly, work from our lab demonstrated that *IDH1* mutation directly causes the G-CIMP phenotype, reduces global 5-hmC through TET2 inhibition, results in hypermethylation of the repressive histone marks H3K9 and H3K27, and blocks differentiation (Turcan et al., 2012). It is important to highlight that widespread loss of 5-hmC is an additional epigenetic hallmark in *IDH* or *TET2* mutated cancers, including melanoma, and that reestablishment of the 5-hmC landscape can suppress tumor invasion and growth in both melanoma cells and a zebrafish model (Lian et al., 2012). Interestingly, *IDH*-associated increases in histone methylation are likely due to 2-HG-mediated inhibition of the Jumonji C (JmjC)-domain-containing histone demethylases (Lu et al., 2012). Still, others have proposed additional mechanisms in *IDH*-mutated cancers such as HIF1a stabilization through PHD inhibition, altered ECM structure due to decreased hydroxylation of collagen, and possible metabolic shifts in NADP/NADPH ratio (Sasaki et al., 2012; Zhao et al., 2009).

Recently, several exciting studies have shed light on novel mechanisms by which *IDH* mutations initiate malignant transformation and how underlying mechanisms may be exploited for therapeutic gain. In mouse models of leukemia and melanoma, IDH mutants accelerated cell cycle transition by activation of the MAPK/ERK pathway and repression of tumor suppressors *CDKN2A* and *CDKN2B* (Chaturvedi et al., 2013; Shibata et al., 2011). Although several studies have noted that *IDH* mutations cause increased colony formation in soft agar and enhanced proliferation, two mouse models for leukemia found that *IDH* mutation primes cells by inducing an MDS- or MPN-like state. However, combining *IDH1* mutants with HOXA9, or *IDH2* mutants with FLT3 or NRAS, was sufficient to initiate transformation (Chaturvedi et al., 2013; Chen et al., 2013a; Xu et al., 2011). This may be cancer-specific though, as mutant IDH2 alone was recently shown to be sufficient to induce sarcoma formation in mice, at least in one model system (Lu et al., 2013). Regardless, the primary mechanism underlying IDH-induced oncogenesis in several model systems is a block in cell differentiation (Pirozzi et al., 2013). Both groups showed restoration of differentiation and increased apoptosis following treatment with IDH inhibitor HMS-101 or Brd4 inhibitor JQ1. Similarly, Losman and colleagues showed that IDH mutant leukemic transformation is specific to the (R)-enantiomer of 2-HG, which can independently promote cytokine independence and block differentiation. Again, this transformation was reversible with IDH inhibitor AGI-5198 (Losman et al., 2013). Lastly, IDH inhibition can reverse novel EMT-associated expression patterns, though a lengthy delay to phenotypic change suggests more stable epigenetic alterations may be to blame (Grassian et al., 2012).

Despite the targeted nature of IDH inhibitors, *IDH* mutation likely unleashes epigenetic marks that are themselves selectable, such as DNA methylation. There is thus interest in the therapeutic potential of 5-azacytidine (5-aza) and decitabine (DAC), due to the complex downstream effects of *IDH* mutations on the cancer methylome. In order to determine if broader epigenetic therapies could “unlock” glioma initiating cells (GIC) from a dedifferentiated state, we treated both wild-type and mutant IDH cell lines with DAC and/or IDH inhibitor AGI-5198. Along with Borodovsky and colleagues, our group found that hypomethylating agents potently induce differentiation, impair colony formation, and suppress *in vivo* growth in IDH mutant cells only (Borodovsky et al., 2013; Turcan et al., 2013). This demonstrates that mutant IDH-induced DNA methylation likely plays a role in maintaining the self-renewal capacity of glioma tumor initiating cells. Similar effectiveness has also been seen in leukemia and chondrosarcoma (Chaturvedi et al., 2013; Chen et al., 2013a; Lu et al., 2013). Interestingly, IDH inhibition using the mutant IDH1 inhibitor AGI-5198 was not nearly as effective, which suggests that broader epigenetic therapies may be necessary to reverse more permanent changes induced by long exposure to mutant IDH. In addition, combination therapy with other IDH affected processes such as histone hypermethylation may have a role and warrant further investigation.

## HISTONE METHYLATION

Histone methylation is a reversible process that takes place at the amino acid side chains of lysine, arginine, and histidine residues. Lysine methylation on histones H3 and H4 is the best characterized and catalyzed by the lysine methyltransferases (KMTs) through the required methyl group donor SAM. All of the KMTs except DOTL1/KMT4 have a catalytically active SET domain and are highly specific to both histone residue and degree of methylation (mono- vs. di- vs. tri-methylation) (Feng et al., 2002; Rea et al., 2000). Generally, methylation at H3K4, H3K36, and H3K79 corresponds to euchromatic or transcriptionally active regions of the genome, whereas methylation at H3K9, H3K27, and H4K20, is associated with heterochromatic regions and gene silencing. In addition, each residue is capable of four methylated states: unmethylated or mono-/di-/tri-methylated. This provides further regulatory diversity in the histone code. For example, H3K4me2/3 is found at TSSs of active genes, whereas H3K4me1 tends to localize to enhancer regions (Greer and Shi, 2012; Kampranis and Tsichlis, 2009).

In contrast, the lysine-specific demethylases (KDMs) work in opposition to the KMTs through the catalytic removal of methylation marks on histone tails. The two families of KDMs responsible are the (FAD)-dependent amine oxidases and the larger JmjC-containing family of α-KG/Fe(II)-ion dependent oxygenases (Shi and Whetstine, 2007). KDM1A/B (LSD1/2) and KDM5A-D (JARID1A-D) catalyze demethylation at H3K4, whereas KDM2A/B (JHDM1A/B) and KDM4A-C (JMJD2A-C) target H3K36, leading to repressed gene transcription at these sites. Alternatively, transcriptional activation is induced in part by demethylation at H3K9 and H3K27 by KDM1A, KDM3A-C (JHDM2A-C), or KDM4A-D and KDM6A/B (UTX/JMJD3), respectively (Varier and Timmers, 2011). Due to the broad and essential nature of these epigenetic marks across the genome, genetic aberrations of histone modifiers have powerful effects on vital cellular processes such as differentiation and cell cycle control, among others.

### Writers (KMTs)

#### EZH2

EZH2/KMT6 is the enzymatic component of the polycomb repressor complex 2 (PRC2), which is responsible for methylation at H3K27 and subsequent gene silencing (Kirmizis et al., 2004). Other essential subunits of the PRC2 complex through which EZH2 interacts include embryonic ectoderm development (EED), suppressor of zeste 12 homologue (SUZ12), and RBAP48/RBBP4. Collectively, these polycomb group (PcG) proteins have been shown to regulate vital cellular processes including differentiation, cell identity, stem-cell plasticity, and proliferation (Margueron and Reinberg, 2011; Shih et al., 2012). As a result, aberrations in any PRC2 component can have powerful physiologic consequences on the cell.

Alterations in *EZH2* were first discovered in breast and prostate cancer, where amplification and overexpression first implied it may function as an oncogene (Kleer et al., 2003; Varambally et al., 2002; Yang and Yu, 2013). This finding was validated both *in vitro* and *in vivo*, with *EZH2* overexpression proving sufficient to drive proliferation in cancer cells and transform primary fibroblasts (Bracken et al., 2003; Croonquist and Van Ness, 2005). Recent sequencing studies have identified numerous mutations of *EZH2* in a variety of leukemias and lymphomas, including follicular lymphoma (FL; 7%–22%), diffuse large B-cell lymphoma (DLBCL; 14%–21.7%), high grade B-cell lymphoma (18%), MDS/MPN (6%–13%), CMML (11.1%), T-ALL, and AML (Abdel-Wahab et al., 2011; Bodor et al., 2011; Capello et al., 2011; Ernst et al., 2010; Grossmann et al., 2011; Lohr et al., 2012; Makishima et al., 2010; Morin et al., 2010; Nikoloski et al., 2010; Ryan et al., 2011; Zhang et al., 2012). Interestingly, frequent missense and truncating mutations were observed, which generated some confusion in the field about whether *EZH2* could possess both pro- and anti-oncogenic functions. Clinically, *EZH2* mutations seem to commonly predict poor prognosis—worse OS/leukemia-free survival (LFS), high-risk IPSS score—especially in myeloid malignancies (Guglielmelli et al., 2011; Khan et al., 2013; Nikoloski et al., 2010). Additionally, *EZH2* mutation associates with *BCL2* rearrangement in FL and germinal center B-cell like DLBCL (GCB-DLBCL) and is notably absent from activated B-cell like DLBCL (ABC-DLBCL) (Beguelin et al., 2013; Morin et al., 2010; Ryan et al., 2011).

One landmark finding that emerged from the wealth of recent sequencing data was the identification of the highly recurrent heterozygous Y641 mutation (Y/F, Y/N, Y/H, Y/C) in FL, DLBCL, and other lymphoid neoplasms (Bodor et al., 2013; Bodor et al., 2011; Morin et al., 2010). Initially thought to be inactivating due to reduced catalytic ability against a short H3-like peptide *in vitro*, Y641 mutant EZH2 exhibited a powerful gain-of-function phenotype when incubated against the entire nucleosomal unit (Sneeringer et al., 2010). In addition, Sneeringer and others have observed a powerful synergy between the wild-type and Y641 forms of EZH2 both *in vitro* and *in vivo*. Whereas EZH2 is very efficient at catalyzing monomethylation of H3K27 but not di-/tri-methylation, EZH2 Y641 shows enhanced ability for di-/tri- methylation at H3K27 (Ryan et al., 2011; Wigle et al., 2011; Yap et al., 2011). Similar findings have also been observed with A677G and A687V mutant EZH2, though these are far less prevalent in cancer (Majer et al., 2012; McCabe et al., 2012a). Since almost all *EZH2* gain-of-function mutations are heterozygous, the overall consequence of these mutations is a cooperative and thereby efficient silencing of genes associated with the repressive H3K27 mark.

Recent studies have shown that mutant EZH2-driven tumors can be effectively targeted with small molecule inhibitors. Knutson and colleagues were the first to describe potent phenotypic effects in lymphoma cell lines, following treatment with the SAM-competitive EZH2 inhibitor (EPZ005687) (Knutson et al., 2012). This inhibitor was highly selective, inducing cell death in mutant EZH2-expressing cells only. As expected, these cells showed global reduction of the H3K27me2/me3 histone mark and significant enrichment of cell cycle gene sets by GSEA. The more recent EZH2 inhibitor GSK126 was also highly selective for mutant EZH2 lymphoma cells *in vivo* and led to increased activation of known EZH2 target genes, such as *TXNIP* and *TNFRSF21* (McCabe et al., 2012b). Although *EZH2* mutation alone may be insufficient to induce development of B-cell lymphoma, new evidence suggests it functions as a master regulator of GCB phenotype through repression of *CDKN1A*, *IRF4*, and *PRDM1* (Beguelin et al., 2013). Due to frequent activation of *EZH2* in lymphoma, these new targeted therapies hold exciting promise in the clinic.

#### SETD2

The major KMT responsible for H3K36 trimethylation is SETD2/KMT3A, which is a novel candidate tumor suppressor gene (TSG) (Edmunds et al., 2008). Gene deletions in clear cell renal cell carcinoma (ccRCC)-derived cell lines are common, reduced expression is seen in breast tumors, and loss is associated with decreased H3K36 trimethylation (Duns et al., 2012; Duns et al., 2010; Newbold and Mokbel, 2010). *SETD2* mutations are quite common in ccRCC (7.4%–11.6%), pediatric high-grade glioma (HGG; 15%), and adult HGG (8%) (Cancer Genome Atlas Research Network, 2013; Dalgliesh et al., 2010; Duns et al., 2010; Fontebasso et al., 2013; Hakimi et al., 2013b; Varela et al., 2011). Almost all of the mutations characterized so far are frameshift or nonsense and therefore truncating, further suggesting *SETD2* may be an important TSG in select malignancies (Hakimi et al., 2013a). Furthermore, Hakimi and colleagues found that *SETD2* mutations were significantly associated with worse cancer-specific survival (CSS) in ccRCC (Hakimi et al., 2013b). Though phenotypic effects of *SETD2* inactivation have not been clarified, recent research showed that *SETD2* loss triggers MSI and can increase genome-wide mutation rates through alterations in H3K36 methylation (Li et al., 2013a; Schmidt and Jackson, 2013).

#### MLLs

The mammalian mixed lineage leukemia (MLL) family of genes encodes a series of active (MLL1–4/KMT2A–D) and inactive (MLL5/KMT2E) KMTs, which have all been implicated in cancer. MLL1–4 are responsible for methylation at H3K4 and share a common core formed by WDR5, RbBP5, Dpy-30, and Ash2L (Varier and Timmers, 2011). Notably, MLL1–2 form a complex along with the menin (MEN1) tumor suppressor and recent evidence shows that H3K27 demethylase KDM6A/UTX can complex with MLL2–4 (Hughes et al., 2004; Yokoyama and Cleary, 2008; Yokoyama et al., 2005).

The earliest known alterations in *MLL* family genes involved frequent rearrangements of *MLL1* at 11q23, with recombination involving more than 40 different partner genes and occurring in 60%–80% of infants with ALL or AML (Dimartino and Cleary, 1999; Pais et al., 2005; Thirman et al., 1993). Since then, several missense and truncating mutations have been identified in *MLL1* in bladder, lung, and breast cancer (Gui et al., 2011; Kan et al., 2010). Although *MLL1* acts as a dominant oncogene in liquid tumors, these new discoveries suggest a different recessive role for *MLL1* in some solid tumors (Krivtsov and Armstrong, 2007). Infrequent truncating mutations have also been noted in *MLL4* in medulloblastoma and head and neck squamous cell carcinoma (HNSCC), suggesting a minor but alternative role for this family member as well (Pugh et al., 2012; Stransky et al., 2011).

Notably, recently, many new mutations have been identified in both *MLL2* and *MLL3*, showing diversity of both mutation and tumor type. *MLL2* and *MLL3* mutations are frequently nonsense or frameshift, resulting in a truncated protein lacking the active SET domain (Morin et al., 2011; Parsons et al., 2011; Pasqualucci et al., 2011b; Pugh et al., 2012; Stransky et al., 2011). Along with *MLL1*/*4*, *MLL2*/*3* mutations suggest a dual role for MLL family proteins in oncogenesis, which may depend heavily on cellular context. In addition, mutations have been discovered in numerous cancers, occasionally at high frequency, including colon (*MLL3*, 14%–17%), DLBCL (*MLL2*, 24%–32%), FL (*MLL2*, 89%), AML, breast, GBM, RCC, prostate, pancreatic, bladder, medulloblastoma, and HNSCC (Balakrishnan et al., 2007; Dalgliesh et al., 2010; Gui et al., 2011; Li et al., 2013b; Lindberg et al., 2013b; Mann et al., 2012; Morin et al., 2011; Parsons et al., 2008; Parsons et al., 2011; Pasqualucci et al., 2011b; Pugh et al., 2012; Sjoblom et al., 2006; Stransky et al., 2011; Vakoc et al., 2009; Watanabe et al., 2011). Unfortunately, functional data remains sparse and the importance of these mutations has yet to be characterized. Watanabe and colleagues made the interesting observation that in colorectal carcinoma, *MLL3* mutations were associated with increased MSI, though no mechanism has been proposed (Watanabe et al., 2011). Regardless, they are intriguing candidates for further study in cancer, especially since MLL family proteins are important regulators of HOX proteins and differentiation (Wang et al., 2009a).

#### *NSD1*/*2*

Histone-lysine N-methyltransferase *NSD2*/*MMSET* was first implicated in cancer as a target for rearrangement [t(4;14)(p16.3;q32)] in 15%–20% of multiple myeloma (MM) patients (Chesi et al., 1998). This translocation results in aberrant upregulation of *NSD2*, which first suggested that it may be an oncogene. Subsequent work has shown that knockdown in MM KMS11 cells leads to apoptosis and re-expression of wild-type NSD2 causes increased proliferation (Martinez-Garcia et al., 2011). Furthermore, overexpression of wild-type *NSD2* is sufficient to transform *NSD2*^-/-^ cancer cells *in vivo* and in mouse embryonic fibroblasts (MEFs) (Kuo et al., 2011). Functionally, interactions with HDAC1/2 and catalytic activity at H3K4 and H4K20 have been proposed, though these may be minor (Marango et al., 2008). It is now clear that the NSD2-catalyzed conversion of unmethylated H3K36 to mono- or di-methylated forms, with concomitant decreases in H3K27me3, is the dominant mechanism driving *NSD2*-associated oncogenic reprogramming (Kuo et al., 2011; Li et al., 2009). In fact, Kuo and colleagues demonstrated that NSD2 SET catalytic activity is required for transcriptional activation at several oncogenic loci (*TGFA*, *MET*, *PAK1*, *RRAS2*). Pathway analyses have also identified the following as significantly altered in mutant NSD2 tumors: TP53 pathway, cell cycle, DNA repair, focal adhesion, and Wnt (Kuo et al., 2011; Martinez-Garcia et al., 2011).

Recently, sequencing projects revealed the presence of a highly recurrent mutation in *NSD2* (E1099K), which is present in 7.5% of pediatric B-ALL and other lymphoid neoplasms (Jaffe et al., 2013; Oyer et al., 2013). These studies showed that NSD2 E1099K leads to enhanced colony formation in soft agar and expected increases and decreases in H3K36me2 and H3K27me3, respectively. This new discovery has exciting therapeutic potential—similar to the activating mutations in *EZH2* described above. Additionally, the related KMT *NSD1* was recently discovered to harbor point mutations in multiple cancers, including HNSCC and AML (Dolnik et al., 2012; Yan et al., 2011). If these mutations prove to be similarly activating, both NSD members will represent completely novel areas of epigenetic regulation through which small molecule targeted inhibition could be useful.

### Erasers (KDMs)

#### KDM6A

Among the first cancer-associated mutations in KDMs that were identified were those in *KDM6A* following sequencing of 1,390 patient tumor samples (van Haaften et al., 2009). Remarkably, *KDM6A* mutations were found to be widespread across both solid and liquid tumors, including AML, chronic myelogenous leukemia (CML), T-ALL, MM, Hodgkin’s lymphoma (HL), TCC, breast, colon, esophageal, pancreas, endometrial, GBM, small cell lung cancer (SCLC), non-small cell lung cancer (NSCLC), and RCC (Dalgliesh et al., 2010; Gui et al., 2011; Mann et al., 2012; Mar et al., 2012; Ross et al., 2013; van Haaften et al., 2009). Since then, *KDM6A* mutations have also been discovered in other tumors such as prostate cancer, medulloblastoma, and adenoid cystic carcinoma (Ho et al., 2013; Lindberg et al., 2013a; Robinson et al., 2012). Although these mutations occur at low frequency in most cancers, *KDM6A* mutations in bladder carcinoma are quite common (20%–29%) (Gui et al., 2011; Poon et al., 2013; Ross et al., 2013). Furthermore, *KDM6A* mutations in bladder carcinoma associate with earlier grade and are inversely correlated with stage (Gui et al., 2011). Therefore, *KDM6A* inactivation may be a powerful driver and early event in bladder oncogenesis. However, whether *KDM6A* mutation status holds significant prognostic value in cancer is yet to be determined.

Despite infrequent inactivation in many cancers, mutation and functional data have established that *KDM6A* is a *bona fide* tumor suppressor gene. KDM6A is a 1401 amino acid protein with several N-terminal tetratricopeptide-repeat (TPR) domains and a single C-terminal Jumonji C (JmjC) domain (Shpargel et al., 2012). Early sequencing efforts revealed that a majority of *KDM6A* mutations are either frameshift or nonsense, and since most occur before the active JmjC demethylase domain, they are most likely inactivating. To test this hypothesis, van Haaften and colleagues re-expressed wild-type *KDM6A* in *KDM6A*-deleted cell lines and observed markedly reduced proliferation (van Haaften et al., 2009). Similarly, we recently showed that cancer-specific missense mutations in the JmjC domain can abrogate this growth suppressive effect and may even contribute to a dominant proliferative phenotype. Furthermore, following overexpression, these tumor-specific mutants exhibited reduced demethylase activity at the repressive chromatin mark H3K27me3 (Ho et al., 2013). Dysregulation of methylation at H3K27 may have important consequences in cancer, as demethylation of H3K27 at HOX genes is required for proper differentiation. Interestingly, it has also been reported that KDM6A binds directly to the HOXB1 locus and that its activation requires KDM6A catalytic activity (Christensen et al., 2007; Morales Torres et al., 2013). In addition to regulation through HOX gene targets, KDM6A catalytic activity also activates RB pathway genes through HBP1 to further influence differentiation and cell cycle control (Herz et al., 2010; Wang et al., 2010).

In addition to contributing to tumor suppression via its catalytic domain, KDM6A also has important demethylase-independent roles in cancer. A recent study found that conditional inactivation of *KDM6A* in a mouse model did not change global levels of H3K27me3, though it did contribute to a MDS-like phenotype and reduced migration of HSCs (Thieme et al., 2013). Furthermore, it is also known that KDM6A can bind to KMTs MLL2–4 to promote H3K4 methylation independent of its catalytic domain and that demethylase-inactive KDM6A is sufficient to induce differentiation (Cho et al., 2007; Issaeva et al., 2007; Lee et al., 2007; Morales Torres et al., 2013). In fact, Wang and colleagues mapped the chromatin occupancy of KDM6A, H3K4me2, and H3K27me3 in primary human fibroblasts and discovered that 62% of KDM6A target genes are enriched for univalent H3K4me2 (Wang et al., 2010). Therefore, KDM6A has at least two independent, yet complimentary, mechanisms for shaping the epigenetic landscape in cancer. Indeed, one study found that inactivating mutations in the catalytic JmjC domain caused increased growth yet also led to simultaneous increases and decreases in H3K27me3 and H3K4me1, respectively (Herz et al., 2010). As a result, it is likely that the *KDM6A*-associated phenotypes in cancer are diverse and are linked to the type and location of each driving mutation.

#### Other KDMs

Although *KDM6A* mutations are the most prevalent and best characterized among the KDMs, several others have been identified as significantly mutated across cancer, albeit at low frequency (Cerami et al., 2012; Gao et al., 2013; Parsons et al., 2011; Pasqualucci et al., 2011b). Uniquely, ccRCC harbors mutations in many of the KDMs, including *KDM1A*, *KDM2B*, *KDM3A*, *KDM3B*, *KDM4A*/*B*, and *KDM5C* (Dalgliesh et al., 2010; Hakimi et al., 2013a; Larkin et al., 2012; Shi et al., 2011). The natural function of these KDMs is still being determined, but these studies and others suggest *KDM1A*, *KDM4A*–*C*, and *KDM5B* are putative oncogenes, whereas *KDM6A*/*B* and *KDM3B*/*C* are tumor suppressors. Also, *KDM2A*/*B* and *KDM5A* seem likely to be both pro- and anti-oncogenic, depending on context (Rotili and Mai, 2011). More recently, Niu and colleagues provided the first *in vivo* evidence that *KDM5C* serves as a tumor suppressor following VHL loss in ccRCC and that cancer-specific mutations were inactivating. Furthermore, they demonstrated that HIF2a binds directly to KDM5C, targeting KDM5C to demethylate H3K4me3 at HIF-repressed gene loci (Niu et al., 2012). It will be exciting to determine if some of these mutations are true oncogenic drivers. Additionally, if gain-of-function mutants are identified in KDM oncoproteins, these may be prime candidates for existing KDM inhibitors or new targeted therapies (Rotili and Mai, 2011).

## HISTONE ACETYLATION

Lysine residues on histone tails may also undergo another form of covalent modification through the addition of an acetyl functional group. This process uniquely results in the neutralization of charge normally associated with lysine residues, which weakens the electrostatic interaction between histones and negatively charged DNA. As a result, it is believed that histone acetylation primarily results in a more “open” chromatin configuration, serving as a “mark” of active gene transcription. Several ChIP-seq studies have now confirmed this, showing localization of acetylated histones at enhancers, promoters, and even throughout the transcribed region of active genes (Dawson and Kouzarides, 2012; Heintzman et al., 2007; Wang et al., 2008). In addition to altering the chromatin state directly, these specific histone “marks” further act to recruit other remodelers containing “reader” bromodomains and tandem plant homeodomain (PHD) fingers (Taverna et al., 2007).

The process of histone acetylation is carried out by the lysine acetyltransferase (KAT) enzymes, of which there are two major classes: Type-A, which are usually found in the nucleus and Type-B, which are cytoplasmic and act on free histones. Dynamic regulation of acetylation is also catalyzed by the histone deacetylase (HDAC) enzymes, which oppose the actions of KATs and remove acetyl groups from histone tails. Interestingly, these enzymes are capable of modifying other non-histone proteins—including p53, Rb, and MYC—and have additional roles as transcriptional cofactors, which has led to many challenges in determining their specific roles in cancer and other disease processes (Dawson and Kouzarides, 2012; Iyer et al., 2004).

### Writers (CREBBP and EP300)

CREB-binding protein (CREBBP) and E1A binding protein p300 (EP300) are structurally distinct from other KATs and have unique broad substrate specificity, including the ability to acetylate all four histones *in vitro.* In fact, both proteins are highly conserved, with 75% similarity across their entire length and 63% homology at the amino-acid level (Iyer et al., 2004; Shiama, 1997). Not surprisingly, many functional similarities exist. Both proteins engage in several diverse functions, including chromatin remodeling via KAT activity, acetylation of association proteins (p53, Rb, E2F), and the ability to act as scaffolds for transcription factors and other transcriptional machinery (Bannister and Kouzarides, 1996; Gu and Roeder, 1997; Nakajima et al., 1997). Despite being some of the earliest epigenetic modifiers identified, their roles in both normal physiology and disease are just beginning to be appreciated.

Both *CREBBP* and *EP300* have long been linked to cancer, though the specific roles they play have been harder to elucidate. Suspicion that *CREBBP* may be a tumor suppressor first arose in the mid-1990s, when heterozygous germline mutations were identified in the setting of Rubinstein-Taybi syndrome, a developmental disorder with an increased prevalence of cancer, including leukemia and lymphoma (Petrij et al., 1995). Around the same time, *EP300* was first shown to bind to the E1A viral oncoprotein, suggesting it may also function as a tumor suppressor (Eckner et al., 1994). Soon after these discoveries, the first genetic alteration of *CREBBP* in cancer was identified in M4/M5 AML subtypes, albeit a rare t(8,16)(p11,p13) translocation that fuses the *MOZ* gene with the N-terminus of *CREBBP* (Borrow et al., 1996; Panagopoulos et al., 2001). Interestingly, reports of *MOZ*-*EP300* translocations do exist, though these events may be exceedingly rare (Lai et al., 1985). Though a few early studies identified low frequency *EP300* mutations in colorectal carcinoma, breast cancer, and gastric cancer, the full spectrum of mutational inactivation of *CREBBP* and *EP300* would not be fully evident until the genomics era.

Genomic and exomic sequencing data from the past several years have revealed that *CREBBP* and *EP300* inactivation via mutation is more widespread and frequent than previously thought. For example, *CREBBP* mutations have now been described in NHL (21%), DLBCL (29%), FL (32.6%), TCC (13%), ACC (7%), and relapsed ALL (18.3%), with *EP300* mutations occurring slightly less frequently in NHL (7%), DLBCL (10%), FL (8.7%), TCC (13%), ACC, and relapsed ALL (Gui et al., 2011; Ho et al., 2013; Morin et al., 2011; Mullighan et al., 2011; Pasqualucci et al., 2011a). Additionally, *CREBBP* and *EP300* are collectively mutated in up to 18% of SCLC (Peifer et al., 2012). Interestingly, mutations in both genes are mutually exclusive, suggesting functional equivalency, at least in part. Additionally, the majority of mutations are heterozygous, indicating that both genes most likely function as haploinsufficient tumor suppressors. In line with this, an earlier mouse study showed that heterozygous *CREBBP* loss led to increased neoplasia over wild-type mice (Kung et al., 2000). In almost all of these studies, mutations strongly clustered in the catalytic KAT domain, several of which exhibit reduced acetyltransferase ability *in vitro* at H3K18 and in the non-histone substrates Bcl6 and p53 (Mullighan et al., 2011; Pasqualucci et al., 2011a; Peifer et al., 2012). Though the targets of CREBBP/p300 are diverse, it seems likely that disruption of acetyltransferase ability can be a main contributor to tumor formation.

Despite the identification and initial functional characterization of these tumor-specific mutations, the physiologic consequences leading to oncogenesis remain obscure. For example, in SCLC, *CREBBP*/*EP300* mutations do not lead to any obvious concerted shifts in gene expression, even though several reported mutations are inactivating *in vitro* (Peifer et al., 2012). Due to the diverse roles of CREBBP and p300, it is possible that some inactivating mutations exert phenotypic consequences through other non-epigenetic mechanisms. For example, a more recent discovery showed that both proteins exhibit acetyltransferase specificity for histones H3 and H4 at double-strand break (DSB) sites, facilitating the recruitment of the SWI/SNF chromatin remodeling complex (Ogiwara et al., 2011). Also, *CREBBP* KAT mutations favor constitutive activity of the Bcl6 oncogene over p53, which may be alone sufficient to promote tumorigenesis. Lastly, paradoxical roles for *CREBBP*/*EP300* have been described recently. For example, *EP300* is actually upregulated in melanoma cell lines, and inhibition of KAT function *in vitro* reduces melanoma tumor cell growth (Yan et al., 2013). Additional experiments will be necessary to help identify functional consequences of these mutations and in what specific contexts these two KATs function as tumor suppressors.

### Erasers (HDACs)

In contrast to acetyltransferases, the 18 member HDAC family is responsible for the removal of acetyl groups from lysine residues on histone tails. Similar to KATs, HDACs have a wide range of protein targets, and are also known to deacetylate nonhistone substrates (Ellis et al., 2009). The HDAC family has four major classes, with class I (nucleus), II (nucleus and cytoplasm), and IV requiring the zinc ion for catalytic activity. Class III (sirtuins) are catalytically active in the absence of zinc and share almost no homology with the other HDACs (New et al., 2012). Notably, several HDACs have been implicated in cancer. Specifically, functional experiments have revealed that these HDACs are pro-oncogenic, with increased apoptosis and reduced proliferation (Class I) or reduced angiogenesis and cell migration (Class II) following specific HDAC knockdown (Ellis et al., 2009). Most importantly, this role is congruent with their acetyltransferase counterparts, the KATs, which have been characterized as tumor suppressors (see above).

In the past decade, much work has been done to develop targeted inhibitors of HDACs, since they are now established oncogenes in many cellular contexts. These HDAC inhibitors (HDACi) work to prevent histone deacetylation, facilitating a more open chromatin configuration and leading to increased gene transcription. Vorinostat, the first FDA approved HDACi, is already in use for select neoplasms, and other more selective HDACi show promising efficacy in early trials at reducing cell growth and increasing apoptosis (Witt et al., 2009). Despite the substantial therapeutic potential for HDACi, there is a need to determine biomarkers for treatment response and resistance.

Sequencing data from the past several years has identified inactivating mutations in several HDACs, which may influence the effectiveness of HDAC inhibitor therapy and predict overall response in patients (You and Jones, 2012). Specifically, mutations have now been reported in *HDAC2* (colon), *HDAC4* (breast), and *HDAC9* (prostate) (Berger et al., 2011; Ropero et al., 2006; Sjoblom et al., 2006). Of these, the recurrent frameshift mutation of *HDAC2* in exon 1 has been the most extensively characterized. This mutation is incredibly common in colon cancer (21%), resulting in a premature stop codon and loss of measurable *HDAC2* expression in 83% of mutant tumors (Ropero et al., 2006). Further, *HDAC2* mutations are enriched in MSI colon cancers (43%) (Hanigan et al., 2008). Importantly, *in vitro* work showed that *HDAC2*-deficient cells were resistant to HDACi via trichostatin A, exhibiting no hyperacetylation at target histones H3 and H4 and no reduction in proliferation compared to wild-type *HDAC2*-expressing cells (Ropero et al., 2006). Recent work has identified the pro-apoptotic gene *APAF1* as a likely target for repression by HDAC2, providing a specific mechanism for both HDACi efficacy and resistance in HDAC2 mutant cells, where HDACi does not appreciably alter *APAF1* levels (Hanigan et al., 2008). Lastly, although there is an important role for using HDAC mutation status as a predictor of HDACi treatment response, Ropero and colleagues have further shown that the *HDAC2* mutation itself can cause changes in gene expression, actively leading to increased levels of multiple pro-tumorigenic proteins (Ropero et al., 2008).

## CHROMATIN REMODELING

In addition to gene regulation via covalent histone tail modifications, the ATP-dependent chromatin remodelers also shape chromatin structure and thereby affect gene expression patterns. Several multi-unit effectors share this responsibility, including SWI/SNF, ISWI, INO80, SWR1, and NURD/Mi2/CDH complexes. In the past several years, protein components of the SWI/SNF complex have been found to be frequently inactivated in cancer, and subsequent work has solidified their status as *bona fide* epigenetic tumor suppressors (Wilson and Roberts, 2011).

### SWI/SNF complex

The SWI/SNF complex consists of one or two mutually exclusive catalytic ATPases (SMARCA2/BRM or SMARCA4/BRG1), a group of conserved core subunits (SMARCB1/SNF5, SMARCC1/BAF155, SMARCC2/BAF170), and other variant subunits (Wilson and Roberts, 2011). Two important SWI/SNF complexes implicated in cancer are the BAF and PBAF complexes, which contain the mutually exclusive ARID1A or ARID1B subunits and PBRM1 or BRD7 subunits, respectively (Reisman et al., 2009; Wang et al., 2013). Collectively, SWI/SNF complexes remodel chromatin through the mobilization of nucleosomes both by sliding and by the ejection/insertion of histone octomers (Saha et al., 2006). Through these mechanisms, the SWI/SNF complexes have powerful effects on transcriptional regulation, serving an important role in development through the coordinate activation and repression of critical gene expression programs. Importantly, specificity is most likely achieved through the unique combinatorial assembly of the SWI/SNF complex, facilitated by the sheer size and diversity of the protein subunit repertoire (Wang et al., 1996).

#### ARIDs

The AT-rich interactive-containing domain (ARID) gene superfamily consists of seven members (ARID1–5), of which the following have now been implicated in cancer: *ARID1A*/*BAF250a*, *ARID1B*/*250b*, and *ARID2*/*BAF200*. Mutations in *ARID1A* are the most widely reported in the literature, with remarkable frequency, first reported in ovarian clear cell carcinoma (OCCC; 50%) and endometrioid carcinoma (30%) (Bosse et al., 2013; Jones et al., 2010; Wiegand et al., 2010). Mutations in other cancers exist, including medulloblastoma, breast, lung adenocarcinoma, ACC, hepatocellular carcinoma (HCC), gastric, pancreatic, and neuroblastoma (Fujimoto et al., 2012; Ho et al., 2013; Sausen et al., 2013; Wu and Roberts, 2013; Zang et al., 2012). Interestingly, the majority of mutations are heterozygous, truncating, and evenly spread along the protein, suggesting a possible role as a haploinsufficient tumor suppressor. Functional studies have confirmed this, noting increased proliferation and colony formation, impaired differentiation, and decreased apoptosis following partial *ARID1A* knockdown (Gao et al., 2008; Luo et al., 2008; Nagl et al., 2007; Zang et al., 2012). Correspondingly, re-expression of *ARID1A* decreases cell proliferation (Zang et al., 2012). In addition, a role in differentiation seems likely, though conflicting data on the specific consequences of *ARID1A* inactivation has been complicated through varying technical approaches and model systems (Wu and Roberts, 2013).

Little is currently known about how *ARID1A* inactivation leads to malignant transformation through SWI/SNF chromatin remodeling, though several intriguing possibilities exist. Both ARID1A and ARID1B provide unique and mutually exclusive specificities for SWI/SNF recruitment to chromatin (Wilson and Roberts, 2011). Interestingly, with ARID1A, this process is at least partially independent of its ARID domain, which binds DNA in a non-specific manner only (Dallas et al., 2000). Instead, ARID1A likely contributes to specific recruitment of SWI/SNF by binding transcription factors and transcriptional coactivator/corepressor complexes, including nuclear hormone receptors (Nie et al., 2000; Trotter and Archer, 2004). In fact, Inoue and colleagues showed that re-expression of *ARID1A* in a breast cancer cell line augments transcriptional activation through glucocorticoid receptors, estrogen receptor, and androgen receptor (Inoue et al., 2002). This specificity is likely due to the presence of several nuclear hormone receptor binding sites near the C-terminus of ARID1A (Nie et al., 2000). In light of this, the impressive prevalence of *ARID1A* mutations in hormone-responsive neoplasms (ovary, breast) is likely to be more than just coincidence.

The clinical utility of *ARID1A* mutation status is currently unclear, due to a limited number of sufficiently powered studies. However, Sausen and colleagues recently found that *ARID1A* or *ARID1B* mutations associate with worse OS in patients with neuroblastoma, and a previous breast cancer study noted that decreased *ARID1A* expression can confer worse prognosis (Mamo et al., 2012; Sausen et al., 2013). Several other intriguing observations have been noted, including a tendency toward co-occurrence between mutations in *ARID1A* and *CTNNB1* (β-catenin) or PI3K-Akt pathway alterations, as well as a mutual exclusivity between *ARID1A* and *TP53* mutations (Bosse et al., 2013). Whether *ARID1A*-associated phenotypes arise through additional epigenetic means, such as reported binding partners HDAC1/2, its ubiquitin ligase activity at H2B K120, or by increased MSI, remains to be determined (Bosse et al., 2013; Li et al., 2010).

Mutations in other ARID family members *ARID1B* and *ARID2* have now been reported, though investigation into their clinical and functional relevance is in its infancy. *ARID1B* mutations occur at moderate frequency in neuroblastoma (7%) and HCC (6.7%), with sporadic mutations identified in breast, gastric, and pancreatic cancers too (Fujimoto et al., 2012; Sausen et al., 2013; Shain et al., 2012; Wang et al., 2011). These mutations are usually frameshift and hemizygous, suggesting it may be a tumor suppressor, like *ARID1A*. More intriguing has been the discovery of *ARID2* mutations in HCC (5.8%–6.5%), with strong enrichment in HCV-associated HCC (14%) (Fujimoto et al., 2012; Zhao et al., 2011). *ARID2* seems to mediate anti-proliferative signaling by binding to IFN-inducible promoters to remodel chromatin in response to interferon signaling. As such, mutations in *ARID2* may render IFN-related immune processes incapacitated in the setting of HCV infection, leading to accelerated tumorigenesis. More recently, mutations in *ARID2* were identified in NSCLC (7.3%), making this gene one of the most frequently mutated in this type of cancer (Manceau et al., 2013).

#### SMARCs

SWI/SNF-related matrix-associated actin-dependent regulator of chromatin (SMARC) genes, also known as BRG1-associated factors, are among the most frequently altered and best characterized chromatin remodelers in cancer. These genes encode several SWI/SNF proteins including the catalytic ATPase subunits (either SMARCA2 or SMARCA4), a group of conserved core subunits (e.g. SMARCB1, SMARCC1, and SMARCC2), and variant subunits (e.g. SMARCE1) (Wilson and Roberts, 2011). Tumor-specific mutations in the following SMARCs are especially common: *SMARCB1*/*SNF5*, *SMARCA2*/*BRM*, and *SMARCA4*/*BRG1* (Shain et al., 2012). Alterations in these critical chromatin remodelers have profound effects on vital processes, such as differentiation, cell proliferation, and metastasis.

*SMARCB1* is inactivated via biallelic alterations—deletion and mutation (truncating, missense)—in nearly all rhabdoid tumors (RTs; 98%), an especially lethal cancer that predominantly affects young children (Sievert et al., 2009; Versteege et al., 1998). Recent exomic sequencing data has identified mutations in other tumors and pre-malignant lesions, such as sarcoma, gastric carcinoma, schwannomatosis, meningioma, chordoma, and hepatoblastoma (Christiaans et al., 2011; Hulsebos et al., 2007; Kim et al., 2013; Kreiger et al., 2009; Mobley et al., 2010; Trobaugh-Lotrario et al., 2009). Mouse models for *SMARCB1* loss have verified a tumor suppressive role for this protein, with homozygous loss leading to frequent formation of lymphomas and RT-like tumors at only 11 weeks, which is notably faster than *TP53*-inactivated models of sarcoma (Guidi et al., 2001; Roberts et al., 2000; Wilson and Roberts, 2011). Interestingly, *SMARCB1*-deleted mice do not develop tumors in the absence of SWI/SNF ATPase SMARCA4 (Wang et al., 2009b), suggesting that oncogenesis following *SMARCB1* inactivation is due to aberrant residual activity of SMARCA4-containing SWI/SNF complexes. Additionally, global gains of H3K27me3 following loss of *SMARCB1* may directly contribute to tumorigenesis, as simultaneous *EZH2* loss protects against transformation. In fact, EZH2 elevation occurs immediately following *SMARCAB1* inactivation, and re-expression of *SMARCB1* evicts PcG proteins from the *CDKN2A* tumor suppressor locus, activating transcription of this important tumor suppressor (Kia et al., 2008; Wilson et al., 2010). This evidence provides both a necessary mechanism and a possible therapeutic target (EZH2) in *SMARCB1*-inactivated tumors.

Further mechanistic insights have yielded many other potential therapeutic avenues in *SMARCB1*-mutated tumors. For example, *SMARCB1* plays a critical role in cell cycle control. Mouse models have shown that coinactivation with Rb or p16 does not accelerate tumorigenesis, and simultaneous loss of cyclin D1 protects against tumor formation (Isakoff et al., 2005; Oruetxebarria et al., 2004; Tsikitis et al., 2005). In this vein, cyclin D1 inhibitors may have surprising effectiveness in these patients. Furthermore, direct binding to Myc has also been reported, along with increased Myc expression following *SMARCB1* loss (Cheng et al., 1999; Tsikitis et al., 2005). Though the efficacy of synthetic lethal approaches in targeting Myc-driven tumorigenesis has yet to be established, these strategies may be worthwhile therapeutic candidates. In addition to cell cycle dysregulation, *SMARCB1* inactivation leads to alterations in cell differentiation, specifically by increasing hedgehog signaling through the transcription factor Gli1 (Jagani et al., 2010). Thus, inhibitors of Gli1 provide a fourth potential target for therapy in *SMARCB1*-inactivated tumors. Lastly, increased RHOA activity and subsequent cell migration was observed following knockdown of *SMARCB1*, though therapeutic possibilities of this mechanism remain unexplored (Caramel et al., 2008).

*SMARCA4*, one of two SWI/SNF ATPase subunits, was first identified as a potential tumor suppressor in NSCLC, where expression is lost in 15%–50% of all tumors (Fukuoka et al., 2004; Reisman et al., 2003). *SMARCA4* mutations have also been noted in 35% of NSCLC cell lines, as well as in medulloblastoma, Burkitt lymphoma, melanoma, HCC, ccRCC, HNSCC, RT, pancreatic, breast, and prostate cancer (Cancer Genome Atlas Research Network, 2013; Endo et al., 2013; Love et al., 2012; Medina et al., 2008; Oike et al., 2013; Shain and Pollack, 2013). The majority of mutations and deletions are homozygous, though loss of only one allele may be sufficient to drive tumorigenesis. For instance, 10% of mice heterozygous for *SMARCA4* develop mammary tumors without spontaneous loss of the second allele (Bultman et al., 2000; Bultman et al., 2008). Functional studies have so far identified dual roles for SMARCA4 in both differentiation and cell adhesion/migration. In embryonic stem cells (ESCs), inactivation of *SMARCA4* leads to defective self-renewal and promotes differentiation, while overexpression enhances the epigenetic reprogramming of fibroblasts into induced pluripotent stem (iPS) cells, possibly through increased OCT4 binding to target genes (Singhal et al., 2010). In addition, SMARCA4 has been shown to promote osteoblast differentiation (Flowers et al., 2009). Alternatively, *SMARCA4* overexpression in cervical cancer cell lines leads to increased ROCK1 and stress fiber formation, which is reversible upon *SMARCA4* knockdown (Asp et al., 2002). Through this latter mechanism, SMARCA4 may serve to modulate cell migration and reduce the potential for invasion.

*SMARCA2*, the other ATPase subunit of the SWI/SNF complex, is also a *bona fide* tumor suppressor with frequent loss of expression via epigenetic silencing and more recently, mutational inactivation (Oike et al., 2013). Mouse models have shown that *SMARCA2* deficiency results in proliferative abnormalities, including increased overall weight and tissue-specific increased growth (Reyes et al., 1998). These mice exhibit increased cell proliferation in the prostate as well as androgen independence (Shen et al., 2008). In a similar lung model, heterozygous or homozygous loss of *SMARCA2* led to increased tumor formation (Glaros et al., 2007). Other studies have proposed another mechanism for enhanced tumorigenicity following *SMARCA2* or *SMARCA4* loss, specifically through the induction of an epithelial-mesenchymal transition (EMT) phenotype via the transcription factor ZEB1 (Matsubara et al., 2013; Sanchez-Tillo et al., 2010). Clinically, *SMARCA2* inactivation may predict poor prognosis, as loss of expression in both NSCLC and HCC correlates with worse OS (Endo et al., 2013; Fukuoka et al., 2004; Reisman et al., 2003). Recently, we identified somatic nonsynonymous mutations in *SMARCA2* (5%) following whole-exome and whole-genome sequencing of 60 patients with ACC (Ho et al., 2013). All of these mutations were located in a region encoding the Helicase C domain, which is involved in regulating gene transcription. Other *SMARCA2* mutations have also been reported in another cohort of patients with melanoma (Nikolaev et al., 2012). Despite epigenetic silencing being the dominant mechanism for *SMARCA2* inactivation, specific mutations may also prove important in the pathogenesis of certain cancers.

#### PBRM1

Mutations in chromatin state regulators are very common in ccRCC, with *PBRM1*/*BAF180* mutations noted in 29%–41% of all ccRCCs (Hakimi et al., 2013a; Varela et al., 2011). In fact, the only gene altered more frequently in this malignancy is *VHL*, a well-characterized driver of these cancers. In addition, lower frequency mutation of *PBRM1* has also been described in many other neoplasms, including DLBCL, HNSCC, chronic lymphoid leukemia (CLL), gastric, pancreatic, and breast cancer (Shain and Pollack, 2013; Xia et al., 2008). Many of these mutations are truncating—frameshift and nonsense—though a sizable number are also missense, evenly distributed across the protein (Hakimi et al., 2013a). It is most likely that these mutations are inactivating, as loss of *PBRM1* expression and gene deletion are noted in several cancers and cell lines (Wang et al., 2012). Further, functional studies have shown that re-expression of *PBRM1* in *PBRM1*-deficient cells induces cell growth arrest in G_1_ phase, specifically through binding of the *CDKN1A* promoter, leading to induction of p21 (Xia et al., 2008). PBRM1 contains six tandem bromodomains that bind acetylated histone, two BAH domains that mediate protein-protein interactions, and a HMG domain to bind nucleosomal DNA, all of which provide functional specificity to SWI/SNF remodelers (Wilson and Roberts, 2011).

Exactly how these mutations affect epigenetic reprogramming has yet to be determined, though they are likely pleiotropic, diverse, and dependent on location and type of mutation. It is clear that *PBRM1* inactivation does have clinical consequences, at least in ccRCC. For example, *PBRM1* mutations have been shown to correlate to worse clinical outcomes (Pawlowski et al., 2013). Also, patients with *PBRM1* inactivation are more likely to present with stage III or IV disease, increased tumor size, low differentiation grade, increased perinephric or lymphatic invasion, and are less likely to have an “incidentaloma” at diagnosis (da Costa et al., 2013; Hakimi et al., 2013a; Pawlowski et al., 2013). In addition, smaller tumors (<4 cm) with *PBRM1* mutations are more likely to exhibit stage II pathologic features. Most interestingly, *PBRM1* and *BAP1* mutations are mutually exclusive in ccRCC, and in a side-by-side comparison, *PBRM1* mutation correlates with improved OS over *BAP1* alterations (Kapur et al., 2013; Pena-Llopis et al., 2013).

#### BRD7

BRD7 is a PBAF-specific chromatin remodeler that is frequently deleted in breast cancer. Recently, low frequency mutations in this gene have been identified in several cancers (Cerami et al., 2012; Forbes et al., 2011). For some time, *BRD7* was believed to be an important SWI/SNF tumor suppressor that functions mainly as a cofactor for p53 to activate oncogene-induced senescence (OIS) (Burrows et al., 2010). In fact, *BRD7* was first identified in a loss-of-function screen for genes required for p53-dependent OIS. Drost and colleagues showed that p53 required BRD7 for OIS—and vice versa—and that p53 directly interacts with the N-terminus of BRD7, upstream of its bromodomain. Furthermore, BRD7 was required for induction of several p53 target genes, including *CDKN1A* and *MDM2*, and was capable of direct binding to their associated promoters (Drost et al., 2010). This mechanism likely explains the reported ability of BRD7 to inhibit cell cycle progression from G_1_ to S phase, though direct binding at the *E2F3* promoter may also facilitate this (Peng et al., 2006).

Though this mechanism is likely important in certain cancers, the diversity of *BRD7* phenotypes may be dependent on the unique molecular architecture found within different tissues. For example, a recent study focusing on epithelial ovarian carcinoma showed that *BRD7* can act as a tumor suppressor independent of p53 activity, possibly by sequestering β-catenin in the cytoplasm. These data were further supported by a β-catenin responsive TCF-reporter assay *in vitro* and the presence of decreased *BRD7* expression in high-grade epithelial ovarian serous carcinoma clinical specimens (Bae et al., 2013). Regardless of the mechanism, overexpression of *BRD7* has powerful tumor suppressive effects in cancer cell lines, resulting in decreased cell viability and reduced invasion/migration, independent of p53 status. Lastly, BRD7 may also influence covalent histone modifications themselves, with knockdown resulting in decreased acetylation at H3K9 and direct binding observed *in vitro* at histone residue H3K14 (Bae et al., 2013; Peng et al., 2006). Future studies are necessary to determine which mutations have functional consequences and whether any associated therapeutic vulnerabilities can be exploited.

## HISTONES

An exciting development in the past few years has been the identification of recurrent mutations in genes encoding the histones themselves. Histone mutations in cancer were first discovered following massive exomic sequencing efforts in colon cancer (*HIST1H1B*, 4%) and NHL (*HIST1H1C*, 7%), though frequencies were low and no recurrent mutations were identified (Morin et al., 2011; Sjoblom et al., 2006). However, a recent pair of pediatric glioma studies revealed highly recurrent heterozygous mutations in *HIST1H3B* and *H3F3A*, which encode histones H3.1 and H3.3, respectively. Collectively, Wu et al. and Schwartzentruber et al. reported common *H3F3A* mutations in diffuse intrinsic pontine glioma (DIPG; 78%) and pediatric GBM (22%–31%), with *HIST1H3B* mutations almost exclusive to DIPG (18%). Most striking is the location of these mutations at amino acid residues K27M and G34 (G/R, G/V), which are at or near critical sites for post-translational modification via methylation and acetylation on the histone tail (e.g. H3K27, H3K36) (Schwartzentruber et al., 2012; Wu et al., 2012). This observation, along with the mutually exclusive and monoallelic nature of the K27 and G34 mutations, led to subsequent investigations into the clinical and biologic consequences of these potentially gain-of-function mutations.

Currently, K27M and G34R/V mutations have been found almost exclusively in pediatric HGGs, including CNS-PNET (11%, G34R), though sporadic mutations at these residues have been recently noted in osteosarcoma (G34W) and MDS (K27N) (Attieh et al., 2013; Gessi et al., 2013; Joseph et al., 2013). In fact, the remarkable prevalence of these mutations in pediatric HGGs has enabled a thorough examination into the clinical significance of these events. For example, despite the mutually exclusive nature of K27M and G34 mutations, which implies a common pathway or biological process, there are important differences. First, several studies have noted that the K27M mutation predicts both younger age and worse OS as compared to G34 and wild-type H3.3 (Chan et al., 2013a; Khuong-Quang et al., 2012; Sturm et al., 2012). In one study, G34 mutations were actually associated with improved OS when compared to wild-type H3.3 (Sturm et al., 2012). Second, *H3F3A* mutations are frequently exclusive with *IDH1* mutations and commonly co-occur with *ATRX*/*DAXX, PDGFRA,* and *TP53* alterations, especially in G34 mutant tumors (Schwartzentruber et al., 2012; Sturm et al., 2012). Overlap between alterations in *H3F3A* and *ATRX*/*DAXX* is particularly interesting, since both have been linked to increased CNVs and alternative lengthening of telomeres (ALT) in tumor specimens (Yuen and Knoepfler, 2013). Third, *H3F3A* mutations predict tumor anatomic location—K27M and G34 mutant GBMs occur in midline and hemispheric areas of the brain, respectively (Chan et al., 2013a; Schwartzentruber et al., 2012). Likewise, a groundbreaking study by Sturm and colleagues revealed distinct epigenetic subtypes of GBM, with K27 and G34 mutant tumor samples exhibiting independent gene expression and methylation profiles (Bjerke et al., 2013; Schwartzentruber et al., 2012; Sturm et al., 2012). Interestingly, these profiles may also explain the anatomic preferences; the G34 mutant transcriptomic signature resembles embryonic regions of the neocortex and striatum, whereas the K27M signature is more similar to embryonic regions of the striatum and thalamus. Lastly, these epigenetic signatures harbor important differences in marker genes, with DNA hypermethylation and associated silencing at *FOXG1* (K27M), *MICA* (K27M), or *OLIG1*/*2* (G34) and elevated expression of *PDGFRA* (K27M) (Bender et al., 2013; Sturm et al., 2012). Collectively, these changes in the methylome and transcriptome define the different H3.3 mutant GBM subtypes, correlate to anatomic tumor locations, and indicate potentially different cell origins or initiation events in tumorigenesis. Further, they provide mechanistic insight into *H3F3A*-induced oncogenesis.

Global DNA hypomethylation and decreases in genome-wide histone methylation suggest that mutant H3.3 functions in a dominant-negative manner (Bender et al., 2013; Chan et al., 2013a; Lewis et al., 2013; Sturm et al., 2012). Several groups have now shown that K27M mutant patient samples and cell lines exhibit globally reduced H3K27 di-/tri-methylation and at many loci, DNA hypomethylation (Bender et al., 2013; Chan et al., 2013a; Lewis et al., 2013). Further, forced overexpression of H3.3 K27M in isogenic GBM cells, 293T cells, and MEFs causes reduced H3K27me2/me3 (but not changes in monomethylation) as well as modest increases in H3K27ac, two features of transcriptionally active chromatin (Bender et al., 2013; Chan et al., 2013b). The major mechanism for loss of histone di-/tri-methylation is K27M-mediated inhibition of the PRC2 complex through direct binding of KMT EZH2 at the catalytic site, as well as binding to PRC2 cofactor SUZ12 (Bender et al., 2013; Chan et al., 2013a; Lewis et al., 2013). In fact, *in vitro* KMT assays revealed that K27M reduces KMT activity by 40%–70% and EZH2 catalytic activity up to 85%, with no loss of PRC2-associated KDM activity (e.g KDM6A/B) (Bender et al., 2013). Somewhat contradictory to this finding, ChIP-seq has revealed that hundreds of gene loci are also enriched for H3K27me3 and EZH2 in K27M mutant tumors or following overexpression of K27M, and analysis of differential expression shows increased gene transcription at these loci (Bender et al., 2013; Bjerke et al., 2013; Chan et al., 2013a). The mechanism underlying these paradoxical changes in H3K27 methylation and associated transcription changes is currently unknown (Chan et al., 2013b).

In contrast to the K27M mutation, broad changes in DNA methylation and histone marks underlying G34R/V induced tumorigenesis are less clear. Further, though investigators have shown that G34R/V mutations disrupt methylation at nearby amino acid residue K36, our understanding of the mechanisms leading to malignant transformation is limited. Despite this, analysis of differential gene expression between H3.3 G34R/V mutant and wild-type tumors has already identified some interesting markers (e.g. OLIG1/2), as stated above. As such, the defining feature of H3.3 mutant tumors is the aberrant activation and repression of numerous genes that uniquely contribute to oncogenesis. Understanding the constellation of changes unique to H3.3 mutants should allow identification of additional subtypes and driver genes. Most exciting is the recent identification of G34-induced activation of *MYCN* in pediatric GBM cell line KNS42 (Bjerke et al., 2013). This well-characterized oncogene is potentially druggable through synthetic lethal means, via aurora kinase A (AURKA), or JQ1 inhibition (Huang and Weiss, 2013). Implementing this strategy in patients harboring *H3F3A* G34 mutations could have immense therapeutic benefit. Similarly, identifying and characterizing other differentially expressed genes in *H3F3A* mutant pediatric gliomas could present new ways of treating this very difficult disease.

## SUMMARY AND PERSPECTIVES

Understanding the role of altered epigenetic states has long been a fundamental goal in cancer research. It is now well-documented that distinct chromatin states are both necessary and sufficient to drive tumor formation, sustain increased cellular growth, and encourage metastatic dissemination (Baylin et al., 2001; Jones and Baylin, 2002). However, the link between classical genetics and cancer epigenomics has only recently been explored. In recent years, a plethora of mutations have been discovered in chromatin modifier genes. Studies now show that these alterations have profound effects on the cancer epigenome, leading to potent oncogenic transcriptional programs.

**Figure 2:**
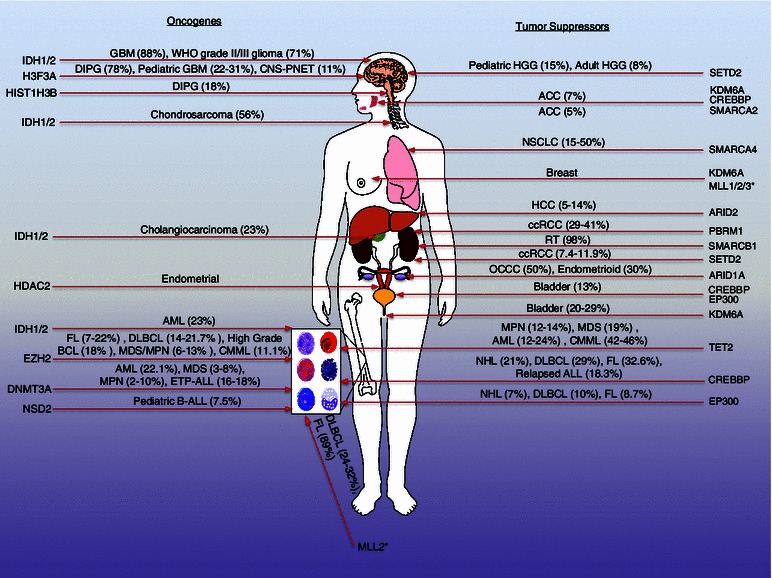
**Frequently mutated epigenetic regulators in human cancer**. Select oncogenes and tumor suppressors implicated in altered cancer epigenomes are shown, along with mutation frequency by tumor histology. *Role in oncogenesis (oncogene vs. tumor suppressor) is either mixed or undetermined

It has become abundantly clear that mutations in epigenetic modifiers are both incredibly diverse and ubiquitous in cancer. In fact, sequencing data has revealed that mutations exist in genes involved in nearly all aspects of epigenetics, including DNA methylation, covalent histone modification, and chromatin remodeling (Fig. 2). Several neoplasms rely on few, yet powerful, mutations in select genes to drive tumorigenesis through an altered epigenome, at least in part (e.g. *IDH1*/*2* in LGG, *MLL2* in FL, *SMARCB1* in RT). Remarkably, some cancers have such a wide range and high prevalence of alterations in chromatin modifiers (ACC, ccRCC, TCC) that they may be driven primarily through epigenetic means. Interestingly, mutations in regulators of DNA methylation have a strong cancer-specific prevalence (glioma, leukemia), whereas genetic alterations in histone modifiers (e.g. *KDM6A*) are widespread across many cancers, though the significance of this is incompletely understood.

Although the field of epigenomics in cancer is relatively new, the identification of driver mutations in epigenetic regulator genes has already led to new prognostic and therapeutic advances. New subtypes of cancer have been identified, or at least attributed to previously unknown genetic alterations, allowing a more nuanced approach to treatment. Further, several new targeted therapies are currently in development and show great promise at reversing phenotypes caused by activating mutations in certain genes (e.g. *IDH1*, *EZH2*). In addition, understanding the epigenetic mechanisms that underlie these alterations has also provided new therapeutic potential, even if the driver mutations cannot be targeted directly (e.g. HDACi, DNMT inhibitors) (Table 1) (Arrowsmith et al., 2012; Foulks et al., 2012; Helin and Dhanak, 2013; New et al., 2012; Plass et al., 2013).

**Table 1:** Selected inhibitors of epigenetic regulators

Substance	Target	Highest clinical status
5-Azacytidine	DNMT	Approved
Decitabine	DNMT	Approved
SGI-110	DNMT	Phase I/II
MG98	DNMT	Phase I
RG108	DNMT	Preclinical
SGI-1027	DNMT	Preclinical
Zebularine	DNMT	Preclinical
EPZ-6438	EZH2	Phase I/II
GSK126	EZH2	Preclinical
GSK343	EZH2	Preclinical
EI1	EZH2	Preclinical
EPZ005687	EZH2	Preclinical
UNC1999	EZH2	Preclinical
Pivanex	HDAC	Phase I/II
Romidepsin	HDAC (class I)	Approved
Vorinostat	HDAC (pan)	Approved
Panobinostat	HDAC (class I/II)	Phase III
Abexinostat	HDAC (class I/II)	Phase II
Belinostat	HDAC (pan)	Phase II
Butyrate	HDAC (class I/IIa)	Phase II
Entinostat	HDAC (class I)	Phase II
Givinostat	HDAC (pan)	Phase II
Mocetinostat	HDAC (class I)	Phase II
Resminostat	HDAC (pan)	Phase II
SB939	HDAC (pan)	Phase II
Valproate	HDAC (class I/IIa)	Phase II
ACY-1215	HDAC6	Phase I/II
PCI-24781	HDAC (class I/II)	Phase I/II
CUDC-101	HDAC (class I/II)	Phase Ib
4SC-202	HDAC (class I)	Phase I
AR-42	HDAC (class I/II)	Phase I
CG200745	HDAC (pan)	Phase I
Tranylcypromine	KDM1A/LSD1	Phase II
ORY-1001	KDM1A/LSD1	Preclinical
AGI-5198	Mutant IDH1	Preclinical
AGI-6780	Mutant IDH2	Preclinical

There is no doubt that we are in the midst of an exciting era in both cancer genomics and epigenetics. Investigations focused on the intersection of these two fields have provided remarkable clarity into numerous aspects of cancer biology and novel mechanisms of oncogenesis. Already, great strides have been made to translate these early discoveries into clinical practice, and the characterization of novel mutations in chromatin modifier genes is rapidly accelerating. With the ongoing development of new epigenetic therapies, it is possible that much improved outcomes, at least in certain cancers, may be possible in the near future. Further, an enhanced understanding of the epigenomic alterations associated with these driver gene mutations could lead to the discovery of novel pathways involved in tumorigenesis, which may themselves possess unique vulnerabilities for therapeutic intervention.

## Abbreviations

ACC: adenoid cystic carcinoma
AML: acute myeloid leukemia
CGI: CpG island
HNPCC: hereditary nonpolyposis colon cancer
HDACs: histone deacetylatases
MSI: microsatellite instability
TCC: transitional cell carcinoma
